# Differences between the intestinal microbial communities of healthy dogs from plateau and those of plateau dogs infected with Echinococcus

**DOI:** 10.1186/s12985-024-02364-4

**Published:** 2024-05-23

**Authors:** Jia Liu, Xiaojie Jiang, Wen Lei, Yuan Xi, Qing Zhang, Huixia Cai, Xiao Ma, Yufang Liu, Wei Wang, Na Liu, Xiongying Zhang, Wanli Ma, Cunzhe Zhao, Bin Ni, Wen Zhang, Yongshun Wang

**Affiliations:** 1 Qinghai Institute for Endemic Disease Prevention and Control, Xining, Qinghai 811602 China; 2 Department of Microbiology, School of Medicine, Jiangsu University, Zhenjiang, Jiangsu 212013 China

**Keywords:** Dog, Intestinal microbial community, Bacteriophage, Echinococcus

## Abstract

**Objective:**

Cystic echinococcosis (CE) represents a profoundly perilous zoonotic disease. The advent of viral macrogenomics has facilitated the exploration of hitherto uncharted viral territories. In the scope of this investigation, our objective is to scrutinize disparities in the intestinal microbiotic ecosystems of canines dwelling in elevated terrains and those afflicted by Echinococcus infection, employing the tool of viral macrogenomics.

**Methods:**

In this study, we collected a comprehensive total of 1,970 fecal samples from plateau dogs infected with Echinococcus, as well as healthy control plateau dogs from the Yushu and Guoluo regions in the highland terrain of China. These samples were subjected to viral macrogenomic analysis to investigate the viral community inhabiting the canine gastrointestinal tract.

**Results:**

Our meticulous analysis led to the identification of 136 viral genomic sequences, encompassing eight distinct viral families.

**Conclusion:**

The outcomes of this study hold the potential to enhance our comprehension of the intricate interplay between hosts, parasites, and viral communities within the highland canine gut ecosystem. Through the examination of phage presence, it may aid in early detection or assessment of infection severity, providing valuable insights into Echinococcus infection and offering prospects for potential treatment strategies.

**Supplementary Information:**

The online version contains supplementary material available at 10.1186/s12985-024-02364-4.

## Background

The mammalian gastrointestinal tract serves as a habitat for a myriad of microorganisms, constituting a heterogeneous and intricate microbial community referred to as the intestinal microbiome [1]. This microbiome encompasses a vast array of microorganisms, encompassing bacteria, viruses, fungi, archaea, and other microbial entities [2]. These microorganisms intricately inhabit the gastrointestinal tract and undertake crucial functions in preserving gut homeostasis, facilitating digestion, contributing to the development of the immune system, and promoting the overall health of the host organism [3].

Phages, widely acknowledged as the most prevalent and diverse biological entities across a multitude of environments including oceans, soil, and the human body, surpass bacteria in sheer abundance, with estimations placing the global number of phage particles in the magnitude of 10^31^ [4]. Characterized as viruses specialized in infecting and replicating within their bacterial hosts, phages manifest a multifaceted relationship with their bacterial counterparts. Functioning as predators, these viral entities can invade and lyse bacterial cells, thereby exerting influence over the abundance and diversity of bacterial populations [5]. Additionally, phages facilitate the horizontal transfer of genetic material between bacteria via a mechanism known as transduction, thus fostering horizontal gene transfer and driving bacterial evolutionary processes [6]. Undoubtedly, phages wield a profound impact on bacterial communities, exerting significant influence in shaping the composition of microbial ecosystems.

Next generation sequencing (NGS) technology has revolutionized our ability to study the microbiome and its association with various diseases. By sequencing the genetic material (DNA or RNA) extracted from microbial samples, researchers can identify and characterize the diverse microbial populations present in a given environment, such as the gut [7].

Cystic Echinococcosis (CE) is a zoonotic disease with a broad range of hosts including dogs, sheep, goats, cattle, and deer, as well as humans [8]. Human infection can occur through direct contact with an infected definitive host or by ingestion of food or water contaminated with tapeworm eggs. The disease is endemic in various regions worldwide and has a wide prevalence in China, particularly in highland pastoral areas [9]. It results in significant morbidity and mortality in both human and animal populations, as well as economic losses due to reduced livestock productivity, veterinary expenses, and public health expenditures [10].

Gut microbiomics encompasses the investigation of the multifarious consortia of microorganisms dwelling within the gastrointestinal tract, encompassing bacteria, fungi, and viruses. Meanwhile, parasites constitute a group of organisms ubiquitously inhabiting the gut milieu [11]. The intricate interplay between these entities assumes a pivotal role in the maintenance of intestinal well-being and the equilibrium of the immune system.

Primarily, parasites can influence the microbial composition of the gut. Empirical research has unveiled that the presence of parasites can induce alterations in the diversity and proportional abundance of gut microflora [12]. Certain parasites may engage in symbiotic relationships with specific microbial communities, thereby precipitating modifications in the microbial composition (e.g. Blastocystishominis) [13]. These perturbations may exert profound repercussions on the host's immune system and overall health.

Secondarily, the composition of the gut microbiome also exerts an impact on parasitic infections. Specific bacterial strains resident within the gut microbiome may manifest antiparasitic properties, thereby diminishing the susceptibility to parasitic infestations through resource competition, the production of antimicrobial agents, or the modulation of the host's immune response (e.g. Entamoeba) [14].

In summation, the interrelations between parasites and gut microbiomics are intricate and bear substantial significance. Delving into these intricacies can augment our comprehension of intestinal health and furnish valuable insights for prospective advancements in the realm of disease prevention and therapeutics, thus opening new vistas for progress in the domains of medicine and biology.

In this study, the focus was on investigating the differences in gut microbial communities between highland dogs infected with Echinococcus and healthy control highland dogs. The study employed viral metagenomics, which involves analyzing the genetic material of viruses present in the samples. By constructing a phylogenetic tree, the researchers aimed to understand the relationships and evolutionary history of the viral communities within the gut microbiota of highland dogs. The findings of this research could contribute to a better understanding of the interactions between host, parasites, and viral communities in the gut ecosystem of highland dogs.

Investigating phage communities within the gut microbiome of infected dogs in the context of Echinococcus infection can help us understand how the presence of the parasite affects bacterial composition and phage-host interactions. By analyzing the phages present, researchers have the potential to identify specific phages that target the bacteria associated with Echinococcus infection, helping to detect or assess the severity of infection early and providing valuable insights into infection with Echinococcus and potential treatment strategies for Echinococcus.

## Methods

### The collection of canine samples

In order to conduct a comprehensive viral metagenomic analysis, a total of 1970 canine fecal samples were collected within the human population's residential activity area. These samples include wild canine feces and domestic canine feces from the local area. The staff wore Level 1 personal protective equipment during the collection process and used disposable chopsticks to sample the canine feces found on the ground. Each canine fecal sample was placed in a 50 mL centrifuge tube and stored at -80 °C for a minimum of 7 days. Each canine fecal specimen underwent scrutiny for the detection of Echinococcus through the employment of Enzyme-Linked Immunosorbent Assay (ELISA) encapsulation.The ELISA protocol involved the use of a canine echinococcosis fecal antigen test kit provided by Shenzhen Bide Biotech. Specifically, 780 fecal samples were obtained from healthy dogs and 170 fecal samples were acquired from dogs carrying Echinococcus in the Yushu Tibetan Autonomous Prefecture, Qinghai Province, China, spanning the period from 2020 to 2021. Additionally, 840 fecal samples from healthy dogs and 180 fecal samples from dogs carrying Echinococcus were collected from the Golog Tibetan Autonomous Prefecture, Qinghai Province, China. For the libraries that tested positive in the ELISA reaction, we utilized the proteome of the class Eucestoda as a database for BLASTx searches to further validate the presence of Echinococcus granulosus. The results indicate that all of the libraries were positive only for Echinococcus granulosus, while other parasites of the same order were negative. This approach suggests that the presence of other related cestode parasites is minimal or absent in these samples (Figure S1). This methodology not only provides a rigorous validation mechanism for the presence of Echinococcus granulosus but also enhances the accuracy of parasitic diagnostics by differentiating between closely related species within the Eucestoda class. The entire sample collection process and all experiments performed in this study were formally approved by the ethics committee of Jiangsu University and the Qinghai Institute of Endemic Disease Control. The collected samples were promptly stored in a refrigerator set at -80 °C to ensure optimal preservation. Notably, sample collection and all related experimental procedures adhered to the ethical guidelines and regulations approved by the ethics committees of Jiangsu University and the Qinghai Institute of Endemic Disease Control and Prevention. In addition, all sample pretreatment activities were carefully performed within the confines of the biosafety level II laboratory of the Qinghai Institute of Endemic Disease Control and Prevention.

### Handling of canine fecal samples

Each fecal sample underwent individual homogenization using a mortar and pestle, after which it was resuspended in 1 mL of Dulbecco's Phosphate Buffered Saline (DPBS). Subsequently, the samples were subjected to rapid freezing and thawing, repeating this process three times on dry-ice. Following the freezing and thawing steps, the supernatants were carefully collected through centrifugation, conducted at 10 min, 15,000 g, and 4 °C. The samples were randomized and grouped into sets of 10, with consideration given to both the geographical area of sample collection and the health status of the dogs. Within each group, approximately 100 μL of supernatant from each sample was carefully transferred to the corresponding sample pool. In this manner, the 1,970 samples were combined to form 197 pools, with an average of 10 samples per pool. To eliminate particles of eukaryotic and bacterial cell sizes, the sample pools underwent centrifugation (20 min, 12,000 g, 4 °C), and the resulting supernatant was subsequently filtered through a 0.45-μm filter [15, 16]. The filtrates were then treated with DNase and RNase enzymes at 37 °C for 60 min to ensure digestion [17–19]. According to the manufacturer's recommendations, all remaining nucleic acids (both DNA and RNA) were isolated using the QIAamp Viral RNA Mini Kit (QIAGEN). Since the nucleic acids are composed of RNA, they were reverse-transcribed into cDNA using a reverse transcriptase kit (SuperScript IV reverse transcriptase) that includes six random primers. Subsequently, different viral templates were used to synthesize dsDNA for constructing DNA libraries. The addition of Klenow fragment polymerase (New England Biolabs) was employed to synthesize the second cDNA strand (dsDNA). Simultaneously, for ssDNA viruses, the ssDNA was converted to dsDNA in this Klenow reaction. The Nextera XT DNA Sample Preparation Kit from Illumina was used to generate 197 double-stranded DNA (dsDNA) product pools.

### Genome sequencing procedures

Subsequently, these sample pools were subjected to sequencing on the Illumina NovaSeq 6000 platform. Each sample pool was sequenced using 250 bp paired-end sequencing and dual-barcode sequencing.

### Bioinformatics analysis

In order to facilitate the utilization of bioinformatics analysis, a total of 197 libraries were constructed and subsequently subjected to the decoding process, which involved the extraction of 250 base pairs from paired-end reads of individual libraries, employing Illumina's proprietary software. The internal analysis pipeline, operating on a 32-bit Linux cluster, was utilized for data processing.

To enhance data integrity, a specific criterion was applied: if bases 5 to 55 of a read were identical, only one random duplicate was retained, and any other duplicates were removed. Subsequently, sequencing results exhibiting subpar quality were trimmed, using a Phred quality score of 10 as the threshold.

The cleaned reads were then subjected to comparison with the internal non-virus non-redundant protein database through DIAMOND BLASTx to eliminate spurious viral identifications [20]. Taxonomic classification of the DIAMOND results was accomplished by parsing the data with MEGAN, and the LCA-assignment technique was executed using default settings.

Furthermore, Geneious Prime v2019.0 (Biomatters Ltd) was employed for the de novo assembly of each viral sequence read [21]. To identify the specific types of viruses present and exclude any erroneous viral sequences, the contigs and singlet sequences were compared to a viral proteome database using BLASTx, with a stringent E-value threshold of less than 10^-5.

The virus BLASTx database employed in the analysis consisted of the NCBI virus reference proteome, in addition to viral protein sequences from NCBI nr FASTA files, categorized based on their annotation taxonomy within the Virus Kingdom. Contigs that did not yield significant hits in the BLASTx search were subsequently compared to viral protein families in the vFam database using HMMER3 to identify more distant viral protein similarities [22, 23].

Lastly, Geneious Prime software was utilized to estimate open reading frames (ORFs) within the viral genome, and the results of the BLASTx searches were integrated with the Geneious Prime analysis to enhance the precision of ORF prediction.

### Virus genome determination and PCR validation

In cases where gaps occurred between contigs within the same viral genome, PCR was employed for bridging purposes. Subsequently, the obtained sequence was subjected to Sanger sequencing to measure its accuracy. The predicted Open Reading Frame (ORF) was then extracted and compared using the BLASTx.

### Analysis of viral communities

The statistical analysis pertaining to the experiment was conducted using Megan v6.21.16 and R v4.2.1. The composition analysis of 197 libraries was standardized and compared using Megan [24]. Subsequently, R v4.2.1 was utilized with the pheatmap and vegan packages to visually represent the structure and abundance of the viral community. Additionally, R v4.2.1, in conjunction with the ggplot2 package, was employed to transform the dynamic changes of the viruses into visual graphics. Statistical significance in the study was determined when the *p*-value was less than 0.05.

### Phylogenetic analysis

In this study, a phylogenetic analysis of the virus's protein sequences was conducted. The analysis involved constructing a phylogenetic tree that included the best BLASTx match of the virus in the NCBI GenBank database and representative sequences from its corresponding family. To predict the viral Open Reading Frame (ORF), the software Genesis Prime and comparisons with the NCBI database were employed. The protein sequences of interest were then compiled using Mega v11.0.13 [25]. Subsequently, a Bayesian inference tree was constructed using MrBayes v3.2.7 [26]. In MrBayes, the phylogenetic analysis was performed on the amino acid sequences using the specified program ("prset aamodelpr = mixed"), which encompasses 10 built-in amino acid models. The number of generations was set to a maximum of 10 million, and the operation stopped when the standard deviation of the final split frequency was below 0.01 [27]. Finally, the resulting phylogenetic tree was visualized and edited using Figtree v1.4.4 and Adobe Illustrator 2020 v26.0.1 to create a visually appealing representation.

### Quality control

To mitigate the risk of nucleic acid contamination within the laboratory setting, sterile double-distilled water (ddH2O) was prepared using high-pressure sterilization. This sterile ddH2O was then subjected to the same processing conditions as the blank control group to ensure consistency. Throughout the entire experimental procedure, standard laboratory preventive measures were strictly adhered to in order to prevent cross-contamination and degradation of nucleic acids. It is important to note that substances in direct contact with the nucleic acid samples do not contain DNase or RNase. Moreover, the dissolution of nucleic acid samples involved the use of an RNase inhibitor and water treated with Diethyl pyrocarbonate (DEPC).

## Results

### Overview of canine virome

In order to investigate the presence of enterovirus communities in high-altitude dogs, a comprehensive collection of 1,970 canine fecal samples was obtained from Yushu and Guoluo prefectures in Qinghai Province. Among these samples, 780 fecal samples were sourced from healthy dogs in Yushu, 170 fecal samples were obtained from dogs carrying Echinococcus in Yushu, 840 fecal samples were collected from healthy dogs in Guoluo, and 180 fecal samples were acquired from dogs carrying Echinococcus in Guoluo.

From these collected samples, a total of 197 libraries were constructed and subjected to metagenomic sequencing using the Illumina NovaSeq platform. This sequencing effort resulted in the generation of 416,769,906 raw reads, with an average GC% of 57.9%.

**Fig. 1:**
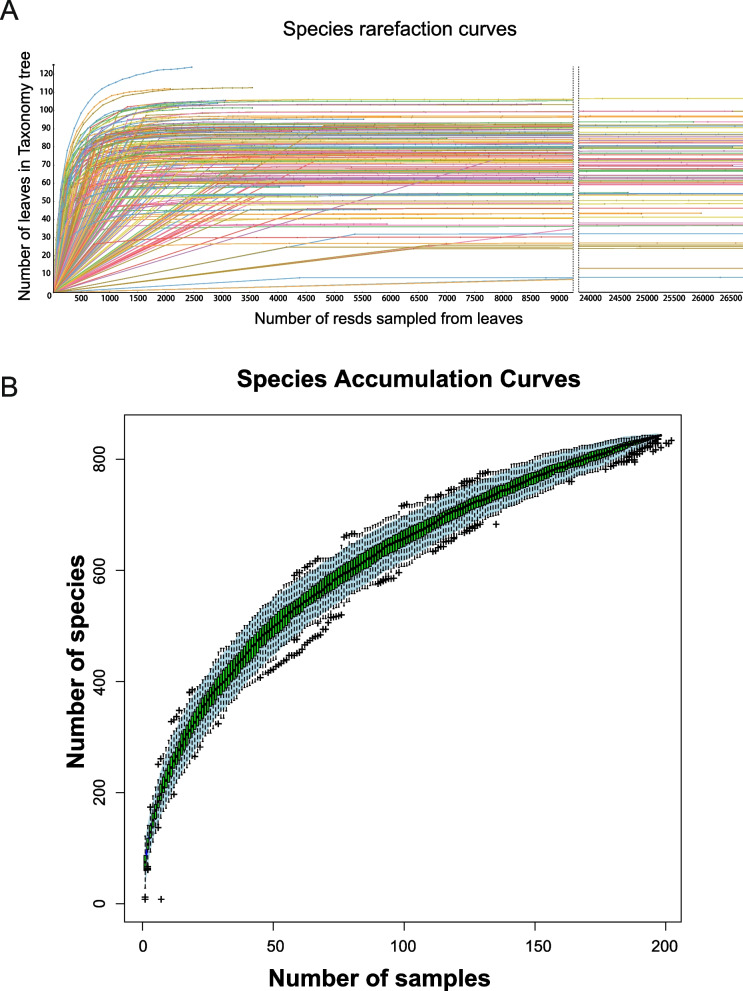
The diversity of viral species in the 197 libraries. **A** Species rarefaction curves were generated by applying a logarithmic scale transformation to the raw data using Megan v6.21.16 software. **B** The accumulation curve illustrates the diversity of viral species in canine metagenomes. Each box plot on the graph represents the richness values of individual samples, with light blue regions indicating the associated 95% confidence intervals

Species richness was assessed using species rarefaction and accumulation curves, which revealed that the viral species observed in the 197 libraries had reached saturation. This suggests that our current sequencing depth has adequately captured all viral species in our samples, and further sequencing is unlikely to increase species diversity (Fig. 1A).

As the number of samples increased, the accumulation curves became smoother, indicating that our sample size was sufficient and representative of our study (Fig. 1B). In total, more than 800 different viruses were identified across the 197 libraries.

**Fig. 2:**
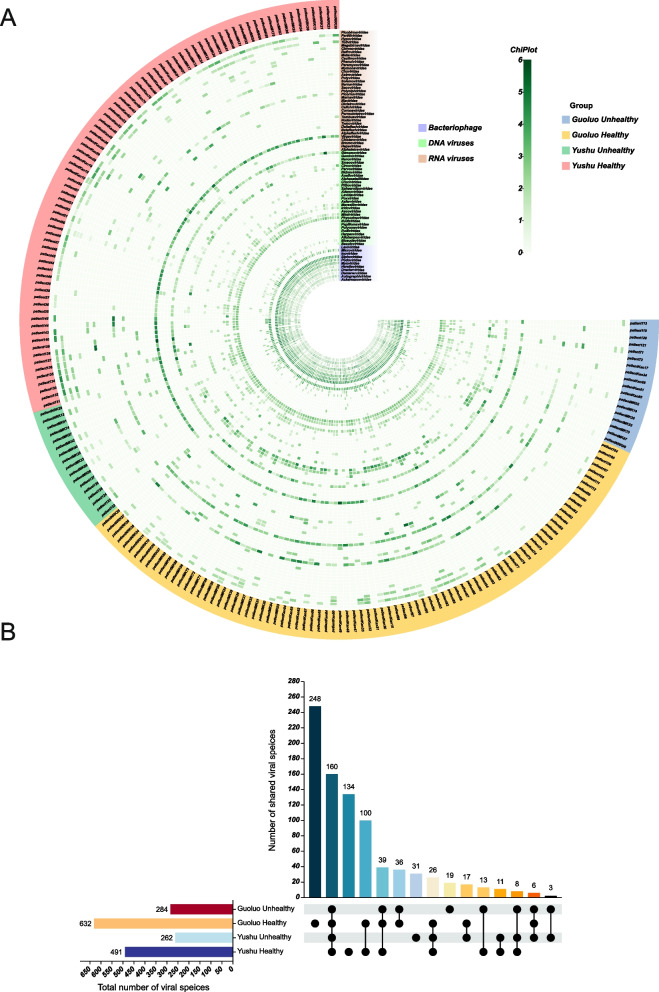
Statistical analysis of enterovirus communities. **A** The heatmap was created to display the nucleic acid types, virus families, sampling area groupings, and health status, which were annotated using different colors according to the provided color legend. **B** The upset plot reflected the number of virus species that are unique or common to each group

A heatmap was generated to examine the variations in viral composition among the 197 libraries. The heatmap was constructed based on the viral families at the family level, the sampled regions, and the nucleic acid types of the viral genome sequences. The data obtained was log-transformed to facilitate visualization. The heatmap revealed the presence of 77 different virus families across the 197 libraries, including 11 phage virus families, 29 DNA virus families, and 37 RNA virus families (Fig. 2A). An upset plot was generated to analyze the virus species at a more specific level, illustrating the count of unique and shared virus species among the four groups. Among these groups, the Guoluo health group exhibited the highest number of virus species, with a total of 632 species. Furthermore, the Guoluo health group exhibited a substantial number of unique virus species, comprising a total of 248 distinct species. It is worth noting that there were 160 virus species levels that were unique to all four groups. Notably, the healthy groups in both regions exhibited a substantially higher number of virus species levels compared to the unhealthy groups (Fig. 2B).

Through the analysis identified, a total of 136 viral genome sequences belonging to eight distinct virus families were identified. *Myoviridae* (*n* = 22), *Podoviridae* (*n* = 1), *Siphoviridae* (*n* = 25), *Schitoviridae* (*n* = 3), *Herelleviridae* (*n* = 1), *Drexlerviridae* (*n* = 6), *Autographiviridae* (*n* = 16), *Microviridae* (*n* = 62).

### Diversity analysis of virus communities

**Fig. 3:**
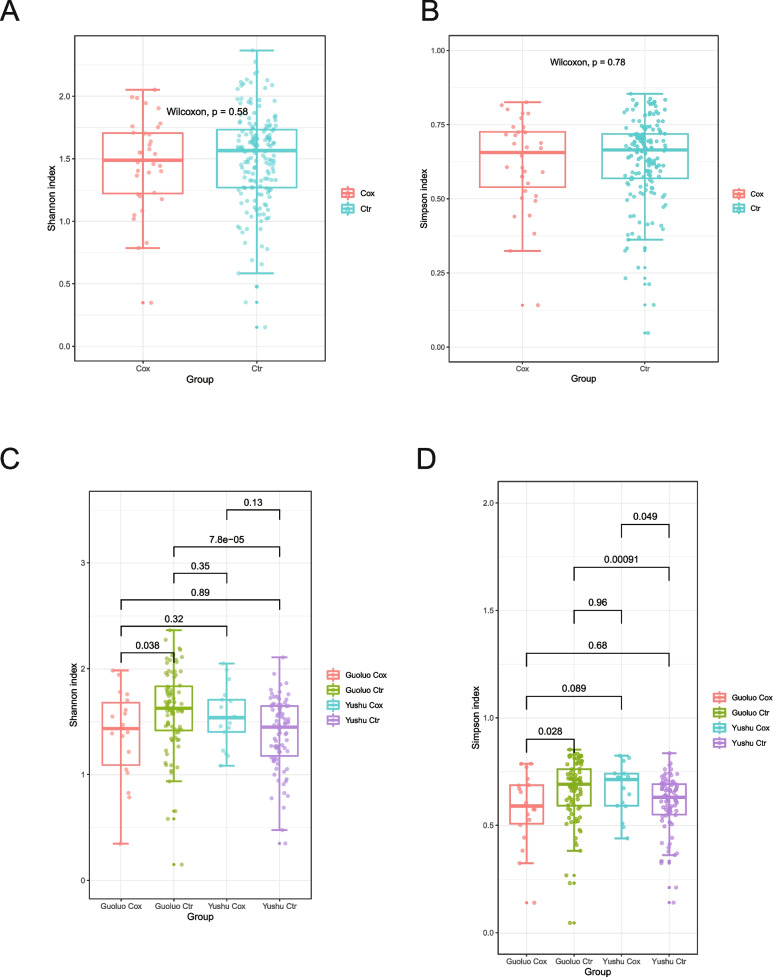
Alpha diversity of enterovirus communities. Prior to comparing viral alpha diversity, the viruses under examination were normalized using Megan. The viral abundance at the family level was quantified using the Shannon index and Simpson index as a measure. The *p*-value was calculated using the Wilcoxon test. Statistical significance was determined when the *p*-value was less than 0.05

**Fig. 4:**
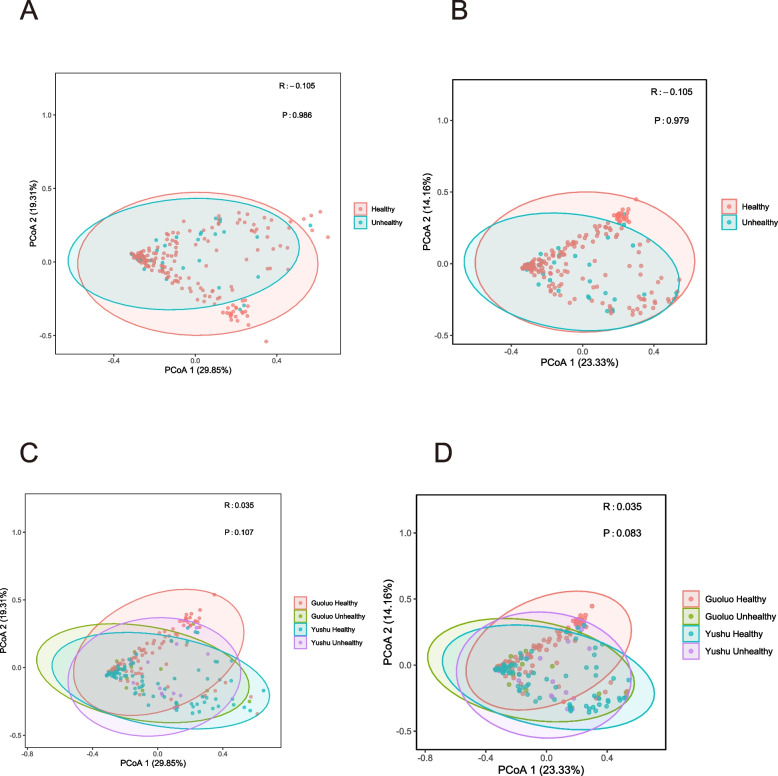
Beta diversity of enterovirus communities. To assess viral beta diversity, the viruses were normalized using Megan before conducting the comparisons. To ensure accurate estimation of beta diversity, we employed both Bray–Curtis and Jaccard metrics. Both analyses conducted in (**A** and **C**) employed the Bray–Curtis index to assess beta diversity. The Jaccard index is used in (**B** and **D**) to analyze beta diversity. Statistical significance was determined when the *p*-value was less than 0.05. A value of R greater than 0 indicated differences between the groups. Statistical significance was determined when the *p*-value was less than 0.05

In order to delve deeper into the variations in the composition of canine GI virus communities, we conducted alpha diversity analysis and beta diversity analysis. These analyses allowed us to explore the diversity within individual virus communities (alpha diversity) as well as the dissimilarity between different virus communities (beta diversity). Initially, we categorized the samples into healthy and unhealthy groups based on the presence or absence of fine-grained echinococcosis. However, the alpha diversity and beta diversity analyses did not yield statistically significant results, as the *p*-values were greater than 0.05 (Fig. 3A and B, Fig. 4A and B). To further refine the grouping, we incorporated regional conditions, resulting in a division into four groups. Upon closer examination, we observed a significant difference in virus communities between the healthy and unhealthy groups in Guoluo, with a *p*-value below 0.05 (Fig. 3C and D, Fig. 4C and D). Additionally, significant differences were observed between the healthy and unhealthy groups in both Guoluo and Yushu, with *p*-values well below 0.05 (Fig. 3C and D). However, the beta diversity analysis still did not yield statistically significant results, as depicted in (Fig. 4C and D).

### Identification and phylogenetic analysis of the order *Caudovirales*

*Caudovirales*, characterized by double-stranded genomes, exhibit significant variations in size, structure, and genomic composition. In the genomes of *Caudovirales*, the Terminase Large Subunit (TERL) serves as a conserved genetic marker for studying their diversity and genetic evolution. In this study, the 197 libraries examined contained a diverse array of *Caudovirales*, including *Siphoviridae*, *Myoviridae*, *Autographiviridae*, and others. To obtain a comprehensive understanding of these *Caudovirales*, the sequences were de novo assembled to obtain 74 *Caudovirales* with complete TERL regions. Subsequently, these sequences were subjected to BLASTx comparison for validation.

**Fig. 5:**
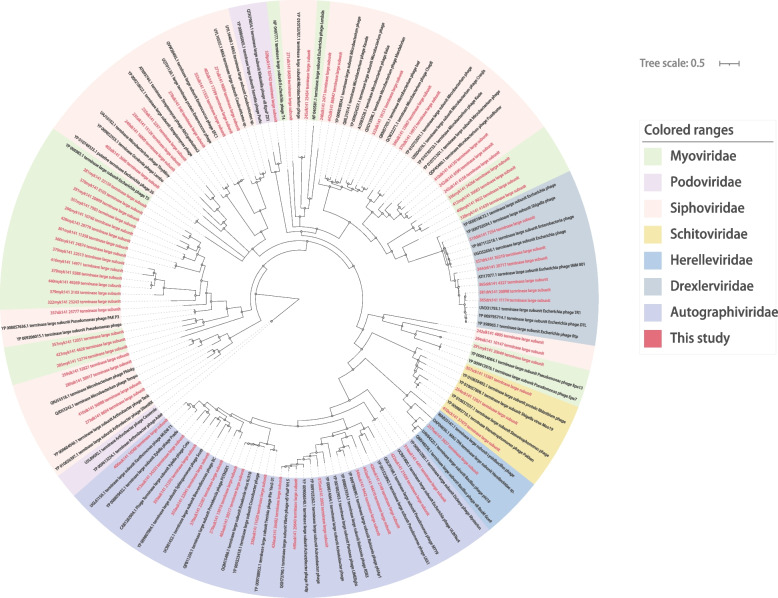
Phylogenetic relationship of the order *Caudovirales*. A Bayesian inference tree was generated based on the complete amino acid sequences of TERL (Terminase Large Subunit) from other members of the *Caudovirales* order. In the provided data, the sequences obtained from this study are represented by the color red. Each scale bar indicates the amino acid substitutions per site

To elucidate the relationship between the newly discovered *Caudovirales* and the known *Caudovirales* in this study, a phylogenetic analysis tree was constructed based on the amino acid sequences of TERL proteins. The phylogenetic tree revealed a high level of diversity among the newly discovered *Caudovirales*. Interestingly, the newly discovered *Myoviridae* and *Siphoviridae* did not form a distinct cluster in the phylogenetic tree diagram. Instead, they appeared in scattered clusters, indicating substantial differences in genome composition between *Myoviridae* and *Siphoviridae* (Fig. 5). This finding underscores the remarkable diversity within the *Caudovirales* order. Such high diversity suggests that *Caudovirales* may play significant roles in the gut community, highlighting their potential importance in this ecosystem.

### Identification and phylogenetic analysis of the family *Microviridae*

**Fig. 6:**
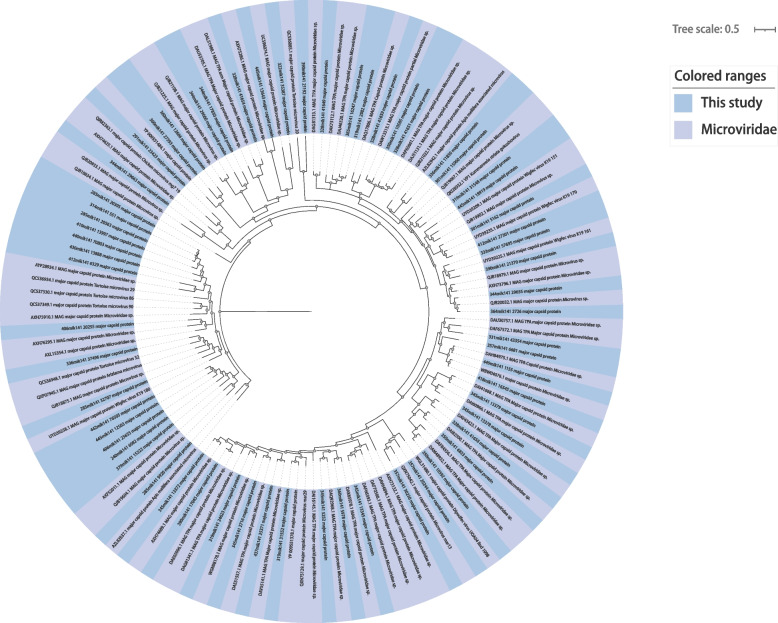
Phylogenetic relationship of *Microviridae.* Bayesian inference trees were constructed using the complete amino acid sequences of MCP (Major Capsid Protein) from the entire *Microviridae* family. In the provided data, the sequences obtained from this study are represented by the color blue. Each scale bar indicates the amino acid substitutions per site

*Microviridae* are a group of small, circular, single-stranded DNA viruses that primarily infect bacteria and are commonly found among ssDNA phages. The Major Capsid Protein (MCP) genes, which are a conserved structural domain, serve as the main region of study for the *Microviridae* [28]. In this study, we identified and de novo assembled 62 sequences with complete MCP regions from the viral reads obtained from 197 DNA libraries of the *Microviridae*. These sequences were then subjected to BLASTx comparison for final verification. To explore the relationships between the newly obtained *Microviridae* and the known *Microviridae*, we constructed a phylogenetic analysis tree using the amino acid sequences of their MCPs. The phylogenetic tree revealed that the newly discovered *Microviridae* share evolutionary connections with the known *Microviridae* and exhibit a high level of diversity (Fig. 6). This high diversity suggests that *Microviridae* may play a significant role in the enteric ecosystem, underscoring their importance in this ecological context.

## Discussion

This study focuses on an in-depth analysis of the gut microbial communities of healthy dogs in highland canines and of highland dogs infected with Echinococcus tapeworm. It is imperative to acknowledge that investigations in this domain hold pivotal significance across various scientific disciplines, encompassing ecology, immunology, epidemiology, and evolutionary biology. The gut microbiota exerts a fundamental influence on host health and adaptation, thus necessitating an in-depth comprehension of their structural composition and functional attributes for the survival and overall well-being of highland canids.

Our research entails a rigorous assessment of viral community diversity within the collected canine fecal samples, aimed at unraveling the intricacies and diversities of the gut microbiota in plateau canids. Through meticulous heatmap visualization and analytical techniques, we have unveiled the substantial richness in the gut microbiota of plateau canines, alongside the presence of a substantial reservoir of DNA viruses and RNA viruses.

Furthermore, the collected samples have been systematically categorized into four distinct groups, predicated on geographic origin and health status, facilitating a meticulous exploration of microbial community disparities between these subsets. This categorization has allowed us to discern unique or shared viral species harbored by each group, offering a preliminary screening mechanism for group-specific viral agents.

Simultaneously, we have conducted a rigorous statistical analysis of the raw data, assessing both alpha diversity and beta diversity parameters. Our analysis has substantiated the existence of statistically significant disparities, notably between the healthy cohort in the Guoluo region and the infected group in the same area. This discrepancy may be attributed to the profound impact of Echinococcus infection on the intestinal microbial community in highland canids. Additionally, we have observed a statistically significant difference between the healthy cohort in the Guoluo region and their counterparts in the Yushu region, potentially attributable to geographical factors.

These findings serve as a critical scientific foundation for a comprehensive exploration of the intestinal microbial community in plateau canids, as well as the repercussions of Echinococcus infection on this ecosystem. Importantly, they offer robust support for the advancement of strategies related to disease prevention and treatment.

In the present study, the identification of *Caudovirales* and *Microviridae* phages as dominant members of the gut microbiome in highland dogs is an interesting finding. *Caudovirales* and *Microviridae* are two major species of phages that infect bacteria. *Caudovirales*, including *Siphoviridae*, *Myoviridae*, and *Podoviridae*, have double-stranded DNA genomes and exhibit diverse structures and genomic compositions [29]. *Microviridae*, on the other hand, are small, circular, single-stranded DNA viruses [30].

The dominance of *Caudovirales* and *Microviridae* in the gut microbiome indicates their prevalence and potential impact on the bacterial populations present. Phages have been known to exert selective pressure on bacterial communities, influencing their composition and dynamics. They can regulate bacterial populations by lysing bacterial cells or modifying their metabolism, thereby affecting the overall structure and function of the gut microbiome [31].

The phylogenetic analysis of phages in this study revealed high diversity within both *Caudovirales* and *Microviridae*. This diversity suggests that phages in the gut microbiome have undergone evolutionary adaptations and may have specific functions within the gut community. Different phage strains within these families may have varying host specificities and target different bacterial species or strains, contributing to the complexity of the gut ecosystem [32].

The interactions between the gut microbiome, the host, and Echinococcus, along with their potential mechanisms and processes, have remained relatively understudied. This area of research holds significant promise for gaining insights into the intricate relationship between viruses, hosts, and the parasitic Echinococcus, a group of tapeworms with the ability to infect both humans and animals. The gut microbiome represents the diverse collection of microorganisms residing within the host's intestinal tract. While numerous studies have focused on host-parasite interactions within the context of Echinococcus infection, the role of the gut microbiome in this interplay remains unclear.

The significant changes observed in the gut microbial composition of canines from the Guoluo region infection with Echinococcus compared to the healthy groups from the same region suggest a potential association between the infection and alterations in the gut microbiome.

The identification of *Caudovirales* and *Microviridae* as important markers in this context indicates that these phage groups may be closely linked to the disease process caused by Echinococcus infection. The differential abundance or activity of *Caudovirales* and *Microviridae* in infected individuals compared to healthy individuals may reflect their potential role in modulating the gut microbial community during the infection.

*Caudovirales* and *Microviridae* phages can interact with bacterial populations in the gut, including pathogens, commensals, and beneficial bacteria. Changes in their abundance or diversity may affect the overall microbial composition and function of the gut microbiome. Therefore, the observed variations in *Caudovirales* and *Microviridae* could potentially contribute to the disease process associated with Echinococcus infection.

We have conducted analytical investigations into the composition of the gut microbiome. Subsequent research endeavors on the interactions between the gut microbiome, the host, and Echinococcus could explore how the gut microbiome interfaces with the host's immune system and the Echinococcus parasite. This exploration might encompass an examination of how the composition of viral groups influences the host's response to Echinococcus infection, encompassing aspects such as the immune response and disease progression. Furthermore, the research could delve into the specific mechanisms through which components of the gut microbiome impact the host's response to Echinococcus infection. This investigation might involve studying the role of viral genes or products in modulating the host's immune system. Additionally, it is worth exploring whether the gut microbiome exerts an influence on the life cycle of Echinococcus, encompassing its growth and reproduction within the host, as this is another critical aspect of study in this field.

It is important to note that while the association between *Caudovirales*, *Microviridae*, and Echinococcus infection is suggested based on the observed changes in the gut microbiome, additional studies are required to establish a causal relationship and determine the functional implications of these phage groups in the disease process. Studying the dynamics and interactions between these phage groups and the bacterial communities in infected individuals can provide insights into the disease progression and potentially open avenues for developing diagnostic or therapeutic approaches targeting the gut microbiome [33].

The present study did not specifically investigate the emergence of new phages and their specificity to highland dogs infection with Echinococcus. It is indeed necessary to conduct subsequent experiments to verify these aspects.

Through techniques such as phage isolation, DNA sequencing, and comparative analysis, it would be possible to assess whether novel phages are present in the gut microbiome of infected highland dogs. Furthermore, testing the infectivity and specificity of these phages against Echinococcus or related strains could provide insights into their potential role in the infection.

By conducting further experiments and analyses, it would be possible to elucidate the dynamics and specificity of phages in relation to Echinococcus infection in highland dogs. This information could contribute to a better understanding of the disease process and potentially lead to the development of novel therapeutic or diagnostic approaches targeting the specific phage-host interactions.

## Conclusion

In conclusion, this study pioneered the exploration of differences in the gut microbial ecosystems of upland canids and canids infected with Echinococcus. The findings propose a potential association between these phage groups and the disease process induced by Echinococcus infection. This study aimed to provide insights into the composition and diversity of the gut microbial community in highland dogs infected with Echinococcus compared to healthy control highland dogs. While acknowledging certain limitations in sample selection, this study introduces novel perspectives for investigating Echinococcus infections and holds promise for the development of innovative therapeutic or diagnostic strategies targeting specific phage-host interactions, pending the identification of specific phages in future research.

### Supplementary Information

**Supplementary Material 1.**

## Data Availability

The viral metagenomic data utilized to corroborate the findings of this study has been submitted and deposited at the National Genomisc Data Center. The quality-filtered sequence reads are accessible in the Sequence Read Archive (SRA) and can be found under the BioProject ID PRJCA015732 and the BioSample IDs SAMC1168982-SAMC1169178. All newly identified genes have been approved by the National Genomisc Data Center and assigned sequence IDs. The serial numbers were C_AA021320-C_AA021455. It is important to note that there are no access restrictions imposed on these datas.

## Abbreviations

CE: Cystic echinococcosis
NGS: Next generation sequencing
ELISA: Enzyme-Linked Immunosorbent Assay
DPBS: Dulbecco's Phosphate Buffered Saline
ORF: Open Reading Frame
DEPC: Diethyl pyrocarbonate
TERL: Terminase Large Subunit
MCP: Major Capsid Protein
