# Supplementary material for: Differences between the intestinal microbial communities of healthy dogs from plateau and those of plateau dogs infected with Echinococcus
Source: Virol J. 2024 May 23;21:116. doi: 10.1186/s12985-024-02364-4 (PMC11112841; doi:10.1186/s12985-024-02364-4)
Supplement: Supplementary file 1 — Supplementary Material 1. [file 12985_2024_2364_MOESM1_ESM.docx]

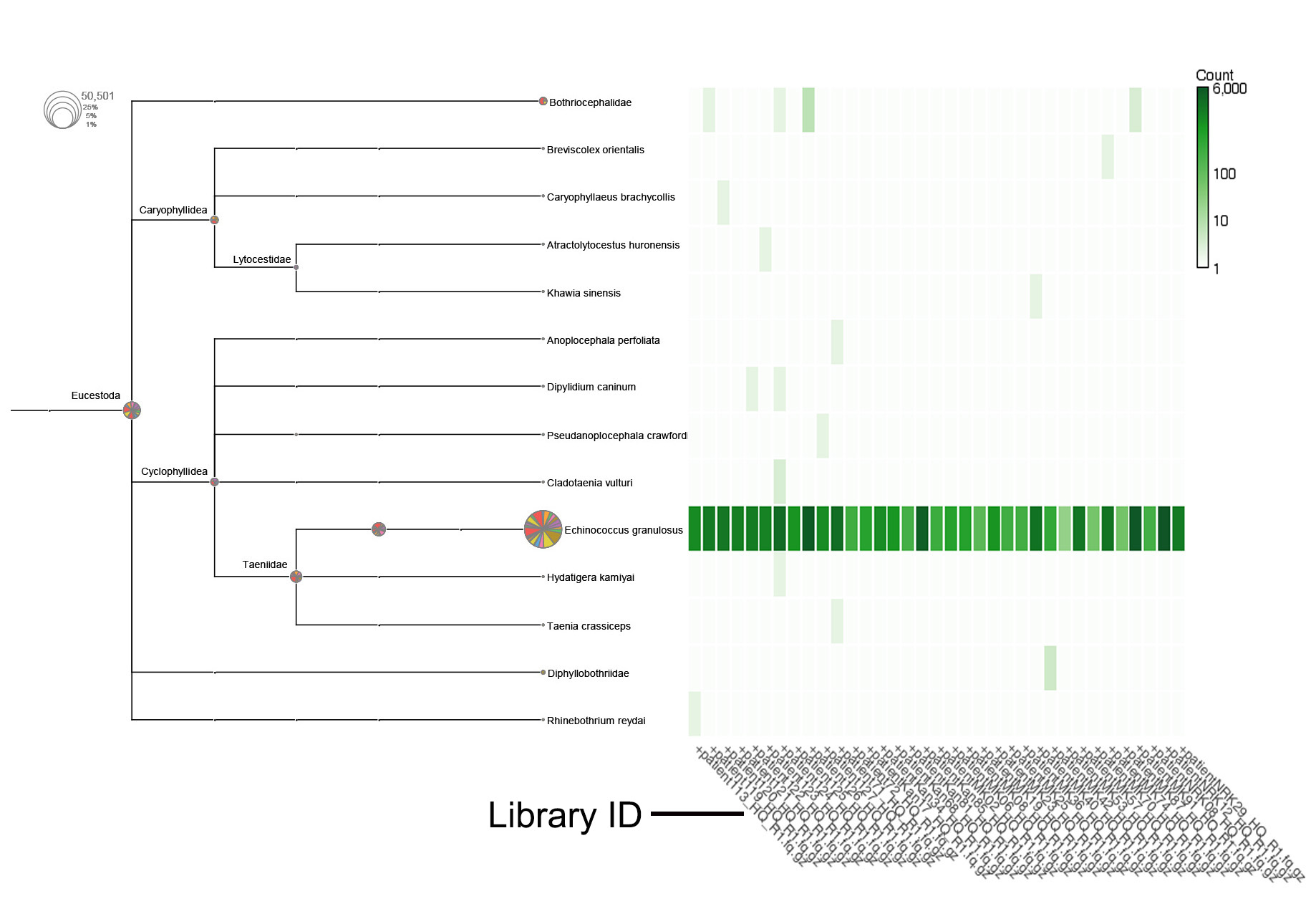


Fig S1. On the left is the name of the specific species of the order Eucestoda. On the right, the heatmap shows the occurrence or abundance of each species across different samples or conditions (Library IDs). The green bars represent the count of observations, scaled logarithmically as indicated by the legend at the top right, from 1 to over 6,000.
